# Fetal Echocardiography Indications and Lack of Association between Abnormal Exams and Advanced Maternal Age: A Cross-Sectional Study - Fetal Abnormal Echocardiography

**DOI:** 10.1055/s-0040-1718445

**Published:** 2020-12-21

**Authors:** Daniela Tarta da Silveira, Cristina Ortiz Sobrinho Valete, Eliane Lucas, Gesmar Volga Haddad Herdy

**Affiliations:** 1 Universidade Federal Fluminense, Niterói, RJ, Brazil; 2 Universidade Federal de São Carlos, São Carlos, SP, Brazil; 3 Universidade da Serra dos Órgãos, Teresópolis, RJ, Brazil

**Keywords:** congenital heart disease, prenatal diagnosis, fetal heart, fetal ultrasonography, cardiopatias, cuidado pré-natal, coração fetal, ultrassonografia pré-natal

## Abstract

**Objective** To analyze the most frequent referrals for fetal echocardiography, including advanced maternal age and its association with abnormal results.

**Methods** We included all pregnant women referred to perform fetal echocardiography (gestational age 22–32 weeks) in 2 health centers in Rio de Janeiro, from June 2015 to June 2016. Advanced maternal age was considered when age was > 35 years at the time of delivery). Referral reasons and results were recorded, according to the Brazilian Fetal Cardiology Statement. Crude and adjusted prevalence ratios were calculated (Poisson regression). We considered *p* < 0.05 as significant.

**Results** A total of 1,221 tests were analyzed. Abnormal fetal echocardiography was observed in 14.82% of the cases. The most frequent abnormalities were interventricular septal defect (6.39%), septal hypertrophy (3.35%) and atrioventricular septal defect (1.14%). Routine exams were performed in 559 women, 289 were referred for advanced maternal age and 373 were referred according to the Brazilian Fetal Cardiology Statement criteria. An obstetric ultrasound suggesting fetal cardiac abnormality, maternal diabetes, increased nuchal translucency, and obstetric ultrasound suggesting a noncardiac abnormality were strongly associated with an abnormal fetal echocardiography. Abnormal results were not more frequent in women with advanced maternal age when compared with the rest of the study group.

**Conclusions** It was observed that routine exams and advanced maternal age referrals were very frequent. Those exams were not associated to fetal echocardiography abnormalities. In this scenario, when the obstetric ultrasound suggests a fetal cardiac abnormality, the fetal echocardiography probably is abnormal. Therefore, obstetric ultrasound is a good screening method.

## Resumo

**Objetivo** Analisar as indicações mais frequentes para realização de ecocardiografia fetal, incluindo idade materna avançada, e a associação destas com exames alterados.

**Métodos** Foram incluídas todas as gestantes que realizaram ecocardiografia fetal na idade gestacional entre 22 e 32 semanas, em 2 centros de referência no Rio de Janeiro, no período de junho de 2015 a junho de 2016. Foi considerada idade materna avançada se no momento do parto a idade materna fosse > 35 anos. As indicações e os resultados dos exames foram registrados, segundo a Diretriz Brasileira de Cardiologia Fetal. Foram calculadas as razões de prevalência brutas e ajustadas através da regressão de Poisson, considerando-se *p* < 0,05.

**Resultados** Um total de 1.221 exames foram analisados. A frequência de exame ecocardiográfico alterado foi 14,82%. As alterações mais frequentes foram defeito do septo interventricular (6,39%), hipertrofia septal (3,35%) e defeito do septo atrioventricular (1,14%). Quinhentos e cinquenta e nove exames foram realizados com indicação de rotina, 289 por idade materna avançada e 373 preenchiam critério de acordo com a Diretriz Brasileira de Cardiologia Fetal. O exame ecocardiográfico alterado foi associado ao ultrassom obstétrico sugerindo cardiopatia fetal, ao diabetes materno, à translucência nucal aumentada e ao ultrassom obstétrico sugerindo alteração extracardíaca. Não foi observada maior frequência de exame ecocardiográfico alterado nas gestantes com idade materna avançada, comparado ao restante da amostra.

**Conclusão** Constatou-se elevada frequência de indicações de rotina, e por idade materna avançada isoladamente, que não foram associados a alterações da ecocardiografia fetal. Em nosso meio, quando o ultrassom obstétrico sugere cardiopatia fetal, é muito provável que a ecocardiografia fetal também seja anormal. Portanto, o ultrassom obstétrico é um bom método de rastreio pré-natal.

## Introduction

Congenital cardiac anomalies occur in ∼ 8 to 18 per 1,000 live births. Research shows that 3 to 4 % of live births have major heart defects that require intervention during the 1 ^st^ year of life. 1 The frequency of congenital heart defects is six times higher than chromosomal defects and four times higher than neural tube defects. 2 3 These anomalies are responsible for up to 10% of deaths in children. 1 4 Fetal diagnosis of cardiac anomalies can improve the prognosis and contribute to the reduction of infant morbidity and mortality, directing expectant mothers to specialized centers. 1 5

There is still no criterion based on scientific evidence to indicate a fetal echocardiography scan for all pregnant women. 6 The American Heart Association published a protocol in 2014 with recommendations for screening of pregnant women considered at high risk. According to this, when the risk of cardiac alteration exceeds 3%, fetal echocardiography should be performed; when the risk is > 2 to 3%, the test should be considered. 7

More recently, the Brazilian Society of Cardiology has published the Brazilian Guideline of Fetal Cardiology, highlighting the clinical conditions that increase the risk of fetal heart alteration and the indications for a fetal echocardiography scan. 8

Although advanced maternal age (> 35 years at the time of delivery) is an important maternal-fetal risk, associated with maternal hypertension, c-section delivery, prematurity, and low birth weight, such factor alone does not constitute risk of fetal heart alteration and, therefore, is not an indication for fetal echocardiography in the two aforementioned guidelines. 7 8 9

The sensitivity of 88.5% and 100% specificity of fetal echocardiography in detecting congenital heart abnormalities should be emphasized, which makes this test an important diagnostic tool. 10

The objective of this study was to analyze the most frequent indications for fetal echocardiography, the alterations found, and if there was an association of advanced maternal age and other factors with abnormal results in two reference centers in Rio de Janeiro.

## Methods

Cross-sectional, analytical study approved by the Research Ethics Committee of Federal Fluminense University, under number CAAE: 51113115.1.0000.5243. Informed consent was waived by the ethics board.

Pregnant women with gestational age between 22 and 32 weeks who underwent fetal echocardiography at Hospital Federal de Bonsucesso or at the Clínica Carlos Bittencourt in Rio de Janeiro from June 2015 to June 2016 were included. Cases of fetal heart poorly visualized were excluded.

Fetal echocardiography was performed by the same pediatric cardiologist, using PHILIPS ENVISOR (Philips, Andover MA, USA) and GE VOLUSON E (GE, Milwaukee WI, USA), with transducers sized 5 to 3.3 Mhz. All tests showed good visualization of the fetal heart, with apical sections of four and five chambers, short and long axis visualization and visualization of the aortic arch and ductus arteriosus. When necessary, color Doppler evaluation was performed. The assessed outcome was an abnormal fetal echocardiography examination (yes/no), suggesting structural or non-structural/functional cardiac alteration. Advanced maternal age was considered if > 35 years at the expected delivery date. The indications for the screening were recorded, and all pregnant women with indications in accordance with the Brazilian Fetal Cardiology Guidelines were considered a risk group.

The data were collected retrospectively from medical records and stored in an Excel spreadsheet (Microsoft Corp., Redmond, WA, USA). Statistical analysis was performed using the Stata version 13.0 program (Stata Corp, LLC, College Station, TX, USA). The sample calculation was performed considering power of 80%, α of 0.05 and 2% difference in proportion between two groups (high and low risk), resulting in 1,044 subjects. The normality of the variable age was tested by the Shapiro Wilk test. The results are presented in medians and interquartile range (IQR). The frequency of indications for the scan, the altered echocardiography, and the alterations detected were calculated, with their respective 95% confidence intervals (95% CIs). Routine indications (regular exams without a specific indication) were analyzed. Differences between proportions were calculated by the chi-squared test. A bivariate analysis was performed with the outcome of the abnormal echocardiographic scan and possible associated variables. Poisson analysis (robust estimation and log-linking function) was performed to estimate crude and adjusted prevalence ratios (CPr and APr). Variables with *p* -value < 0.20 were included to calculate the adjusted prevalence ratios. For final analyses, *p* < 0.05 was considered significant.

## Results

A total of 1,340 scans were performed; 119 were excluded due to poor visualization of the fetal heart. Thus, 1,221 results were analyzed. The average maternal age was 32 years old (IIQ 27–36). The frequency of altered echocardiography was 14.82% (181/1,221). Considering the Brazilian Fetal Cardiology Guidelines, 373 tests (30.54%) fulfilled the indication criteria, and, of these, 142 (38.06%) were abnormal. On the other hand, 848 exams (69.45%) did not meet risk criteria, and, of these, 39 (4.59%) were abnormal ( *p* < 0.001). Advanced maternal age was the isolated indication for 289 tests (23.67%). Of these, 31 results (10.72%) were altered. Routine indication was registered in 559 exams.

Among the group of pregnant women who met the criteria according to the Guidelines ( *n* = 373), the most frequent indications were an obstetric US suggesting extracardiac alteration (26.2%), maternal diabetes (18.5%), and monochorionic twinning (12.6%). Abnormal results were found more frequently when obstetric US suggested fetal heart disease in 82.35% of the cases. The most frequent indications and frequency of abnormal results are found in Table 1 .

**Table 1:** More frequent indications for fetal echocardiography and frequency of abnormal results, according to the Brazilian Guideline of Fetal Cardiology

Indication	n	Abnormal results	%	95% CI
Obstetric US suggesting fetal cardiopathy	34	28	82.35	65.46–93.23
Chromosomal defects	20	14	70	45.72–88.10
Maternal Diabetes	69	22	31.88	21.17–44.20
Obstetric US suggesting extracardiac alteration	98	31	31.63	22.60–41.80
Increased Nuchal translucency	27	8	29.62	13.75–50.18
Cardiac rhythm disorders	12	3	25	5.48–57.18
Monochorionic twinning	47	4	8.51	2.36–20.37
Others	66	32	48.48	35.99–61.11

The most frequent changes were interventricular septal defect, in 6.39% (78/1,221), septal hypertrophy, in 3.35% (41/1,221), and atrioventricular septal defect, in 1.14% (14/1,221). The prevalence and crude prevalence ratio of fetal echocardiographic alterations were calculated according to risk factors ( Table 2 ).

**Table 2:** Prevalence and crude prevalence ratio (CPr) of fetal echocardiographic alterations according to risk factors

Risk factor	n	%	Abnormal result (%)	CPr	*p* -value
Obstetric US suggesting fetal cardiopathy					
Yes	34	2.78	82.35	7,004	< 0.001
No	1,187	97.22	12.88	1	
Chromosomal defects					
Yes	20	1.64	70.00	5,034	< 0.001
No	1,201	98.36	13.40	1	
Maternal Diabetes					
Yes	69	5.65	31.88	2,310	< 0.001
No	1,152	94.35	13.80	1	
Obstetric US suggesting extracardiac abnormality					
Yes	98	8.02	31.63	3,092	< 0.001
No	1,123	91.98	13.35	1	
Increased nuchal translucency					
Yes	27	2.21	29.62	2,044	0.019
No	1,194	97.78	14.48	1	
Cardiac rhythm disorders					
Yes	12	0.98	25.00	1,696	0.295
No	1,209	99.01	14.72	1	
Monochorionic twinning					
Yes	47	3.84	8.51	0.922	0.823
No	1,174	96.15	15.07	1	
Maternal age alone					
Yes	289	23.66	10.72	0.666	0.029
No	932	76.33	16.09	1	

In the multivariate analysis, the factors associated with higher frequency of altered fetal echocardiography ( Table 3 ) were maternal diabetes, fetal US suggesting cardiac or extracardiac alteration, and altered nuchal translucency. Advanced maternal age was not maintained in the model ( *p* -value 0.072).

**Table 3:** Adjusted prevalence ratio (Apr) of abnormalities in fetal echocardiography and associated risk factors

Risk factor	APr	95% CI	*p* -value
Obstetric US suggesting fetal cardiopathy	6,144	4,548–8,302	< 0.001
Maternal Diabetes	3,508	2,378–5,176	< 0.001
Increased nuchal translucency	3,260	1,774–5,992	< 0.001
Obstetric US suggesting extracardiac abnormality	2,190	1,555–3,085	< 0.001

## Discussion

The present study points to the excess of fetal echocardiography tests performed in pregnant women with advanced age without other comorbidities (23.87%). Although this factor constitutes a maternal-fetal risk, it does not constitute risk of fetal heart alteration. This screening was performed without recommendations supporting it. In the present study, as an isolated factor, a lower prevalence of echocardiographic alterations (CPr 0.66) was observed, not justifying its indication for screening; this recommendation was also published in other articles. 9 10 11

The frequency of abnormal results was 14.82%, comparable to the result of Stümpflen et al., 12 who reported 14.9% frequency of altered results. The authors studied pregnant women between 18 and 28 weeks who agreed to have the scan performed. 12 Persico et al. 13 studied risk pregnancies referred for chorionic villus biopsy and observed 11.6% of altered results. A national study in pregnant women without risk detected a frequency of 2.5% of altered tests. 14 The occurrence of altered results may vary depending on the indications and gestational age when the scan was performed. It is noteworthy that the present study was cross-sectional, and the patients were not followed up in the postnatal period; all scans were performed during pregnancy.

Considering the altered results in the high-risk group, according to the Brazilian Fetal Cardiology Guidelines, the frequency was 38.06%, while in other indications it was 4.59%, a significant difference that highlights the importance of the adequate indication for fetal echocardiography. On the other hand, Nayak et al. found a high frequency of abnormal results in the group considered low risk, but no difference between high and low risk groups according to the Pediatrics Council of the American Society of Echocardiography. Of interest, the authors reported 26 exams who failed to detect fetal abnormalities on level-2 ultrasound (for poor window, operator error and other reasons). Those exams in other centers probably should have been considered “obstetric ultrasound suggesting a fetal cardiac abnormality.” This particular result limits the recommendation that fetal echocardiography should be done in all pregnant women, irrespective of risk factors. 15 Other studies have found a high frequency of abnormalities in low-risk groups, which raises the need for detailed methodological assessment for proper comparisons. 16 17 We emphasize the need to follow recommended guidelines to optimize available resources.

In the risk group, the most frequent indication was obstetric US suggesting extracardiac alteration followed by maternal diabetes and monochorionic twinning. Meyer-Wittkopf et al., 18 in 2001, reported the history of congenital heart disease in the family as the most frequent indication (44.5%). Friedberg et al. 19 also reported history of congenital heart disease in the family as the most frequent indication (22%), followed by maternal diabetes (18%) and obstetric US suggesting heart disease (13%). The authors mention that indications for fetal echocardiography have changed over the years as obstetric US has been more accurate, and the high availability of fetal echocardiography may induce indications.

The most frequent echocardiographic change was the interventricular septal defect (6.39%), followed by septal hypertrophy (3.35%) and atrioventricular septal defect (1.14%). Ozkutlu et al. 17 and Sainz et al. 20 found similar results. In Porto Alegre, a population-based study with low-risk pregnant women showed interventricular communication as the most frequent alteration, followed by heart rhythm disorders and fetal hypertrophic heart disease. 14 In our study, it is worth noting that maternal diabetes was the second most frequent indication in pregnant women at risk; septal hypertrophy, a functional change, is associated with this condition. Maternal diabetes is a risk factor for increased left ventricular mass, difficulty in left ventricular relaxation and systolic dysfunction. 21 22

Factors associated with fetal echocardiographic alterations were obstetric US suggesting fetal heart disease (APr = 6,144), followed by maternal diabetes (APr = 3,508), altered nuchal translucency (APr = 3,260), and obstetric US suggesting extracardiac alterations (APr = 2,190). This result is in accordance with the literature. 17 23 It is important to stress that we analyzed the most frequent factors to obtain association estimates in our environment. Due to the study design, no direct risk estimates were obtained. Due to the frequency of the altered factors and scans, we opted for CPr and APr estimates. It is known that in this circumstance, the association estimation by odds ratio may overestimate the association. 24

This study has limitations. The sample was obtained in two centers where fetal echocardiography was performed, and the study results may not reflect the reality of the city of Rio de Janeiro. In the period studied (2015 and 2016), according to the Superintendence of Health Surveillance, 90,539 and 83,057 live births were recorded, respectively (mean 86,798); thus, the present sample represented 1.4% of live births in the period. 25 Also, as a cross-sectional research, no further information on neonatal echocardiography examinations were collected.

## Conclusion

From the results, it was found a high number of scans that did not meet the indication criteria of the Brazilian Fetal Cardiology Guidelines. Special attention should be given to the advanced maternal age, which alone does not constitute fetal risk for cardiac alteration and should not be considered an indication for fetal echocardiography. In our environment, when obstetric US suggests fetal heart disease, it is likely that the fetal echocardiography examination does the same, which reveals the high quality of obstetric US screenings.
